# Neurosyphilis: An Uncommon Diagnosis in the Antibiotic Era

**DOI:** 10.7759/cureus.82383

**Published:** 2025-04-16

**Authors:** Aimal Khan Danish, Muhammad Ijaz Khan, Anwar Ali, Umar Khan

**Affiliations:** 1 General Medicine, University Hospitals Sussex NHS Foundation Trust, Chichester, GBR; 2 Medicine Unit, Khyber Teaching Hospital, Peshawar, PAK; 3 Internal Medicine, University Hospital Kerry, Tralee, IRL; 4 Respiratory Medicine, University Hospital Kerry, Tralee, IRL

**Keywords:** neurology, neurosyphilis, penicillin, std, syphilis

## Abstract

Neurosyphilis results from infection of the nervous system by *Treponema pallidum*. The diagnosis of neurosyphilis is overlooked because of its rarity. It can manifest as early or late symptoms after primary infection with syphilis, with symptoms lying on the spectrum of asymptomatic early neurosyphilis, syphilitic meningitis, meningovascular syphilis, general paresis, and tabes dorsalis. In this report, we present a case of neurosyphilis presenting with acute confusion and agitation.

## Introduction

Cases of neurosyphilis are uncommon compared to the pre-penicillin era. Although early-stage neurosyphilis presents as asymptomatic meningitis, it can present with meningismus, headache, and cranial nerve palsies [1]. Meningovascular syphilis, interposed between early and late forms, involves small and medium-sized arteries in the central nervous system [1-9]. Late neurosyphilis develops decades after the primary infection [1-6]. Common late manifestations are general paresis characterized by psychosis, depression, personality change or progressive dementia, and tabes dorsalis involving gait ataxia and Argyll Robertson pupils [1].

This case report aims to highlight neurosyphilis as a potentially reversible cause of acute confusion and agitation in patients, even in the absence of a known history of syphilis or risk factors. Early recognition and prompt treatment can result in complete recovery and prevent long-term complications.

## Case presentation

A 76-year-old gentleman was referred to our emergency department by his general practitioner, with acute-onset confusion and aggressive behaviour for the last five days. He also had a fall and hit his head in the bathtub. On arrival, as he was unable to give an appropriate history due to confusion, a collateral history was obtained from his next of kin. He had no recent history of fever, cough, chest pain, seizure, or lightheadedness, and he had no past medical history of note, including any sexually transmitted infections. No regular medications and drug allergies were noted. He was a farmer by profession and normally independent in activities of daily life.

At presentation, his blood pressure was 135/80, heart rate was 84 beats/minute, respiratory rate was 20 breaths/minute, temperature was 36.5°C, and SpO_2_ was 99% on room air. His chest was clear, and heart sounds were normal on auscultation. Abdominal examination was normal as well.

On neurological examination, his Glasgow Coma Scale score was 13, and his pupils were noted to be asymmetrical, right 3 mm and left 2 mm, with normal light reflex. No nystagmus was noted. He had normal tone and power (5/5) in all four limbs. Deep tendon reflexes were symmetrical and within normal limits. Sensory examination revealed intact light touch, pinprick, and proprioception across all dermatomes. No cerebellar signs were noted at presentation. The patient had no neck stiffness or photophobia, and Kernig’s and Brudzinski’s signs were negative.

Initially, a septic screen was performed. His full blood count, renal function test, liver function test, C-reactive protein, serum electrolytes, bone profile, and thyroid function test were all normal. Blood glucose was normal. A chest X-ray and urine dip were also found to be normal. Urine culture showed no growth. CT of the brain and angiogram were normal. MRI of the brain (non-contrast) demonstrated bilateral small vessel white matter disease. There was no evidence of infarction, hemorrhage, mass effect, or meningeal enhancement. His vasculitic screen was negative. Human immunodeficiency virus (HIV) antigen/antibody, hepatitis B surface antigen, and anti-hepatitis C virus serologies were negative. The cerebrospinal fluid (CSF) sample was non-hemorrhagic. Cell differential and biochemistry are outlined in Table 1. The CSF polymerase chain reaction results are outlined in Table 2.

**Table 1:** Cerebrospinal fluid cell differential and biochemistry.

	Results	Normal ranges
White blood cells	69/mm^3^	Less than 5/mm^3^
Glucose	2.6 mmol/L	2.5–4.4 mmol/L
Proteins	186 mg/dL	15–60 mg/dL

**Table 2:** Cerebrospinal fluid polymerase chain reaction report.

	Results
Cytomegalovirus	Not detected
Enterovirus herpes simplex virus 1	Not detected
Herpes simplex virus 2	Not detected
Human herpesvirus 6	Not detected
Human parechovirus	Not detected
Varicella zoster virus	Not detected
Escherichia coli	Not detected
Haemophilus influenzae	Not detected
Neisseria meningitidis	Not detected
Listeria monocytogenes	Not detected
Streptococcus agalactiae	Not detected
Streptococcus pneumonia	Not detected
Cryptococcus neoformans/gatti	Not detected

No CSF culture growth was noted after 72 hours. The CSF sample was also analyzed for *Treponema pallidum* serology, and the result was consistent with recent/active syphilis infection. The culture was positive for syphilis total antibody, rapid plasma reagin (RPR), and *Treponema pallidum particle agglutination* (TPPA). This led to the diagnosis of neurosyphilis.

After the confirmation of neurosyphilis as the cause of the patient’s symptoms, he was commenced on benzylpenicillin 2.4 g intravenously every four hours for 14 days, as recommended by the local microbiology department. The patient showed progressive clinical improvement during the 14-day course of intravenous benzylpenicillin and returned to his baseline functional status by the time of discharge. At six months, a repeat lumbar puncture demonstrated normal CSF cell counts and protein levels, and syphilis serology was negative, confirming resolution of the infection.

## Discussion

Decline in the cases of neurosyphilis was made possible with the availability of antibiotics [2]. Currently, neurosyphilis commonly co-exists with HIV infection [3]. Neurological symptoms of syphilis after primary infection may be evident immediately or may take decades to appear [6]. The early manifestation of syphilis includes asymptomatic meningitis or may include headache, meningismus, and nerve palsies [1]. Late symptoms include general paresis and tabes dorsalis [1]. CSF examination in neurosyphilis typically demonstrates elevated protein and lymphocytic pleocytosis, along with reactive serological tests for *T. pallidum*, such as RPR and TPPA, even in patients with no known history of prior syphilis [5]. Elevated white blood cells and proteins in the CSF of our patient pointed toward the diagnosis of neurosyphilis, along with the positive serology. Early diagnosis and treatment are key to a good neurological outcome in neurosyphilis [1]. Although benzylpenicillin is the gold standard for the treatment of neurosyphilis, ceftriaxone has also been found to be equally effective [4]. The patient in this case was treated with intravenous benzylpenicillin and showed full clinical recovery. Normalization of clinical symptoms and CSF findings may take 6-24 months, but a fourfold drop in serum RPR within 6-12 months is considered a reliable marker of successful treatment and may predict CSF recovery, reducing the need for repeat lumbar punctures [7]. Normalization of CSF findings noted in the follow-up CSF examination in this case confirmed the resolution of infection with no residual neurology.

## Conclusions

Neurosyphilis is an uncommon condition to be diagnosed in the era of antibiotics; however, it can still occur in cases of untreated syphilis infections. It should be considered in patients presenting with acute confusion and agitation with no other obvious cause, especially patients with a previous history of syphilis infection. Treatment with antibiotics should be started following the diagnosis of neurosyphilis, as it was noticed in this case that it responds well to treatment. Prognostic outcome depends on early diagnosis and prompt treatment with antibiotics. Benzylpenicillin is the gold standard for the treatment, and patients show full clinical recovery with normal follow-up tests.
